# Advances and Challenges in Pediatric Sepsis Diagnosis: Integrating Early Warning Scores and Biomarkers for Improved Prognosis

**DOI:** 10.3390/biom15010123

**Published:** 2025-01-14

**Authors:** Susanna Esposito, Benedetta Mucci, Eleonora Alfieri, Angela Tinella, Nicola Principi

**Affiliations:** 1 Pediatric Clinic, Department of Medicine and Surgery, University of Parma, 43126 Parma, Italy; benedettamucci@gmail.com (B.M.); ele.alfieri95@gmail.com (E.A.); angela.tinella82@gmail.com (A.T.); 2 (Previous)Università degli Studi di Milano, 20122 Milan, Italy; nicola.principi@unimi.it

**Keywords:** biomarkers, organ dysfunction, pediatric sepsis, cell blood count, C-reactive protein, procalcitonin, lactate, ferritin

## Abstract

Identifying and managing pediatric sepsis is a major research focus, yet early detection and risk assessment remain challenging. In its early stages, sepsis symptoms often mimic those of mild infections or chronic conditions, complicating timely diagnosis. Although various early warning scores exist, their effectiveness is limited, particularly in prehospital settings where accurate, rapid assessment is crucial. This review examines the roles of clinical prediction tools and biomarkers in pediatric sepsis. Traditional biomarkers, like procalcitonin (PCT), have improved diagnostic accuracy but are insufficient alone, often resulting in overprescription of antibiotics or delayed treatment. Combining multiple biomarkers has shown promise for early screening, though this approach can be resource-intensive and less feasible outside hospitals. Predicting sepsis outcomes to tailor therapy remains underexplored. While serial measurements of traditional biomarkers offer some prognostic insight, their reliability is limited, with therapeutic decisions often relying on clinical judgment. Novel biomarkers, particularly those identifying early organ dysfunction, hold potential for improved prognostic accuracy, but significant barriers remain. Many are only available in hospitals, require further validation, or need specialized assays not commonly available, limiting broader clinical use. Further research is needed to establish reliable protocols and enhance the clinical applicability of these tools. Meanwhile, a multifaceted approach that combines clinical judgment with existing tools and biomarkers remains essential to optimize pediatric sepsis management, improving outcomes and minimizing risks.

## 1. Introduction

Pediatric sepsis remains a significant global public health concern, affecting approximately 25 million children annually and resulting in mortality rates ranging from 4% to 50% depending on the clinical severity, risk factors, and geographic variability [1,2]. The burden is particularly high among children under five, with many fatalities occurring within the initial 48–72 h of treatment due to refractory septic shock or multi-organ failure [3]. This early and rapid progression underscores the critical need for prompt identification and intervention in pediatric sepsis cases. Early recognition and rapid initiation of treatment are essential to prevent the escalation to severe multi-organ dysfunction and death. Weiss et al., in a retrospective analysis of children with severe sepsis and septic shock, demonstrated that mortality risk increases with each hour’s delay from sepsis recognition to antibiotic administration [4]. Specifically, a delay of more than three hours before initiating the first appropriate antimicrobial therapy was associated with a significantly elevated risk of mortality in the pediatric intensive care unit (PICU), with odds ratios of 4.84 (95% confidence interval [CI], 1.45–16.2) and 4.92 (95% CI, 1.30–18.58), respectively.

Sepsis induces a multifaceted host response involving an initial proinflammatory surge characterized by widespread vasodilation and endothelial dysfunction, leading to reduced blood pressure and impaired tissue perfusion, which often culminates in multi-organ failure; this is frequently followed by a phase of immunosuppression, further complicating management and underscoring the critical need for early and accurate diagnosis [2]. Pediatric sepsis has important differences from adult sepsis, including age-specific variability of vital signs, developmental age-dependent immune function, and differences in pediatric-specific comorbidities, epidemiology, and outcomes [1]. Therefore, clinical presentation and therapeutic approach are significantly different. This explains why criteria used in adults for diagnosis and treatment of sepsis cannot be applied to children, especially the youngest [1]. In the attempt to overcome this problem, in recent years, consensus criteria for pediatric sepsis and septic shock have been prepared and validated. However, despite efforts to standardize pediatric sepsis identification, current criteria fall short of facilitating early detection. These criteria primarily identify children who already exhibit life-threatening dysfunctions across multiple organ systems (respiratory, cardiovascular, coagulation, and neurological), signaling that sepsis’s complex biological processes have already triggered significant clinical manifestations [5]. Consequently, these criteria are inadequate for screening at-risk children or enabling the early identification of those with suspected sepsis. Furthermore, they often overestimate sepsis incidence by including children with other diseases presenting similar symptoms, such as common acute respiratory infections—the most frequent reason for pediatric emergency department visits. This misclassification leads to unnecessary antibiotic prescriptions, a critical issue due to the resultant antimicrobial resistance, increased drug-related adverse events, extended hospital stays, and elevated healthcare costs. Additionally, existing criteria identify sepsis presence but do not predict disease progression, leaving morbidity and mortality risks unclear and failing to guide the urgency and nature of therapeutic interventions. Moreover, these criteria have primarily been validated in hospitalized children, limiting their applicability in community settings.

To address these limitations, a range of early warning scores and screening tools, including both traditional and novel approaches, have been introduced in clinical practice to better distinguish benign infections from those with a high risk of sepsis development. These tools are the same as those usually used in adults with suspected or diagnosed sepsis but, because of the already cited differences in clinical manifestations of sepsis between children and adults, they may lead to different results in younger patients. For better use in children, they have been studied in children admitted to the hospital or visited in prehospital settings [6,7,8]. Concurrently, there has been substantial progress in identifying biomarkers with the potential to refine sepsis diagnosis, monitor disease evolution, and determine the need for antibiotic therapy, thereby providing clinicians with critical information beyond diagnosis alone [9,10]. The integration of these clinical tools and biomarkers has shown promise in improving pediatric sepsis management, reducing adverse outcomes, shortening hospital stays, and lowering antibiotic usage. However, the clinical effectiveness of these interventions varies, with some studies indicating mixed results.

In this narrative review, we evaluated the role of different clinical prediction tools and commonly used biomarkers in the diagnosis and management of pediatric sepsis. To conduct this review, we systematically analyzed articles published from 2000 to February 2024, using the PubMed database as our primary resource. The literature search employed keywords such as “sepsis”, “septic shock”, “children”, “diagnosis”, “biomarker”, and “early warning”, combined using Boolean operators (“AND” and “OR”) to refine the search and capture the most relevant studies related to pediatric sepsis diagnosis and prognosis.

## 2. Clinical Prediction Tools

Pediatric early warning scores (PEWS) represent the most basic clinical prediction tools designed to identify signs of critical illness and deterioration, including sepsis, in pediatric patients [11]. These manual tools require healthcare providers to evaluate a child’s symptoms and clinical indicators, assigning a score that reflects the severity of deviation from normal values. A score above a set threshold indicates a higher risk of serious illness, potentially pointing to sepsis. Typically, PEWS assessments include vital signs, such as heart rate, blood pressure, oxygen saturation, and respiratory rate, combined with findings from physical exams and relevant medical history. However, PEWS systems vary significantly in their design, predictor variables, outcome measures, modeling methods, and validation processes [11]. Some studies even use different sepsis definitions, making direct comparisons challenging. Additional predictive factors, such as prior diagnoses, current medications, healthcare encounters prior to the emergency department (ED) visit, and lab test results, can sometimes be incorporated to improve prediction accuracy. Although mortality and morbidity are commonly used outcomes for PEWS validation, other factors, such as medication use, hospital stay duration, and disease progression, have also been considered [12].

The diversity of variables included in PEWS contributes to inconsistent performance results, with many PEWS effectively identifying critical conditions but lacking a consensus on which are the most reliable in practice. This variability suggests that finding a universally effective PEWS is unlikely. The following two UK studies exemplify the disparities in PEWS effectiveness: Lillitos et al. [13] assessed the sensitivity and specificity of the Brighton and COAST PEWS for predicting hospital admissions and significant illness. They reported high specificity (>90%) but low sensitivity (32% for hospital admission and 44% for serious illness), indicating limited effectiveness in excluding the need for admission or identifying serious conditions. In contrast, Romaine et al. [14] found more promising results. Comparing the National PEWS with six other UK PEWS in 11,449 febrile children presenting to EDs, they showed that all PEWS performed well in identifying patients needing critical care and those at risk of sepsis-related death, with AUC values between 0.91 and 0.95 for critical care admission and 0.95 and 0.99 for sepsis-related mortality. However, the scores only moderately discriminated for hospital length of stay (AUC: 0.69–0.75). The National PEWS and bedside PEWS were the most accurate overall (AUC: 0.90 for both).

Despite the utility of PEWS in hospital ED settings, their effectiveness in prehospital settings, where rapid identification of critical illness is paramount, is limited. A UK-based study tested the Liverpool quick Sequential Organ Failure Assessment (LqSOFA) and National PEWS in general practice settings [15]. These tools, though validated for hospital use, showed suboptimal sensitivity and specificity in predicting hospital admission within two days of a general practitioner visit. LqSOFA achieved a sensitivity of 30.6% (95% CI 21.8–41.0) and specificity of 84.7% (95% CI 83.7–85.6), whereas the National PEWS had a sensitivity of 81.0% (95% CI 71.0–88.1) but a specificity of only 32.5% (95% CI 31.2–33.8). Using LqSOFA would miss most children needing critical care, while National PEWS would classify a significant number of non-severe cases as requiring hospital admission, leading to unnecessary hospitalizations, increased healthcare costs, and overuse of antibiotics. These findings illustrate that manual PEWS alone may not be sufficient for managing suspected sepsis in children presenting to EDs. Clinical judgment and biomarker data should complement PEWS to provide a more accurate risk assessment.

A promising approach to enhancing PEWS involves integrating sepsis screening tools into electronic health records (EHRs), which overcomes many limitations of manual assessments [16,17]. Automated tools embedded in EHRs allow for real-time tracking and analysis of vital signs and laboratory results, reducing the chance of errors due to oversight and allowing continuous patient monitoring. Automated tools notify clinicians when a child’s score indicates a risk of a life-threatening condition, facilitating timely intervention. They also enable dynamic therapy adjustments if the patient’s condition improves, potentially reducing or eliminating unnecessary antibiotic use. Studies have shown the advantages of these automated tools. For instance, Eisenberg et al. compared an automated sepsis screening tool to traditional manual PEWS in detecting sepsis and septic shock, finding that the automated tool achieved higher sensitivity (84.6%, 95% CI 77.4–90.2) and specificity (95.1%, 95% CI 94.9–95.2) compared to the traditional PEWS (sensitivity: 64.6%; specificity: 91.1%) [18]. This improvement correlated with a reduced length of stay in both hospital and PICU settings for patients in the automated tool group.

Cost-effectiveness is another benefit of automated tools over manual PEWS. Toews et al. reported a 21.2% reduction in median cost per patient with automated monitoring, from USD 6454 (IQR: USD 968–21,697) to USD 5084 (IQR: USD 802–16,618) [19]. These findings suggest that automated sepsis screening could improve clinical outcomes while lowering costs, making them a valuable asset in pediatric sepsis management.

Table 1 shows the comparison of PEWS and their applications in sepsis diagnosis.

## 3. Biomarkers in Pediatric Sepsis Management

Biomarkers serve as measurable, accurate, and reproducible indicators of biological status under both normal and pathological conditions. In sepsis—a complex condition resulting from a dysregulated immune response to infection, often leading to multisystem organ dysfunction [20]—biomarkers can be valuable tools to assist clinicians in diagnosis, risk stratification, and treatment decisions [21]. In managing pediatric sepsis, challenges include diagnosing bacterial infection, differentiating sepsis from non-severe infections, evaluating morbidity and mortality risk, and determining the timing and type of intervention. Although no single biomarker meets the criteria of an ideal marker (~100% sensitivity and >85% specificity), several biomarkers can contribute to a more rational and targeted approach to sepsis management in children [22]. The following sections detail the characteristics and clinical roles of commonly used biomarkers, assessing their effectiveness in pediatric sepsis management (Table 2). Each biomarker discussed offers valuable insights but is limited in scope. For optimal use, biomarkers should be considered alongside clinical judgment and, where possible, used in combination to enhance diagnostic and prognostic accuracy in pediatric sepsis.

### 3.1. Traditional Biomarkers

#### 3.1.1. Blood Culture

Blood culture (BC) remains the only diagnostic test capable of identifying the specific pathogen causing sepsis, enabling targeted antibiotic therapy [23,24,25,26]. However, it is not ideal for early sepsis detection or ongoing monitoring due to its long turnaround time and limited sensitivity, often leading to delays in appropriate treatment. On average, the time-to-positivity (TTP) in pediatric sepsis cases is 12 h (IQR: 8–17 h; range: 0–109 h), though it can extend to 48–72 h in some cases [23]. As a result, nearly all children with suspected sepsis receive empirical antibiotics, leading to overtreatment in those without true sepsis. Highly sensitive and targeted polymerase chain reaction (PCR) methods can reduce TTP, but these are not universally available and do not provide antibiotic sensitivity data. Low-colony-count bacteremia, common in pediatric cases, further limits BC sensitivity. Kellogg et al. found that 60% of bacteremic children had ≤10 CFU/mL, with 23.1% showing ≤1 CFU/mL, classifying these as low or extremely low bacteremia [25]. Proper sample volume is crucial for reliable BC results yet obtaining the recommended 1–1.5 mL for infants or 7.5 mL for older children is challenging in clinical settings, especially in younger infants, often leading to false-negative results [26].

#### 3.1.2. Complete Blood Count (CBC) Parameters

The CBC and its components—white blood cell (WBC) count, absolute neutrophil count, neutrophil-to-lymphocyte ratio (NLR), platelet count, and others—are low cost and widely accessible. However, these parameters are nonspecific markers of inflammation and are influenced by various conditions, limiting their use in sepsis diagnosis. Studies show overlapping CBC values in children with sepsis, healthy children, and those with non-septic conditions, rendering them ineffective in distinguishing sepsis. Generally, these markers have high specificity but low sensitivity, typically not exceeding 20% [27]. The immature-to-total neutrophil ratio (I/T ratio) can provide more reliable information, with sensitivity up to 70% for detecting sepsis [28]. Combining these markers with others may improve diagnostic accuracy, though interpretive variability across laboratories remains a concern. Although high WBC and elevated I/T ratios have been linked to a poorer prognosis, their prognostic utility is limited [29].

#### 3.1.3. C-Reactive Protein (CRP)

C-reactive protein (CRP) is an acute-phase reactant produced by the liver in response to cytokines such as IL-6, IL-1, and TNFα, elevating in various inflammatory states, including bacterial, viral, and fungal infections [30,31,32,33,34,35,36,37,38]. CRP levels rise within 8–12 h post-stimulus, peaking around 48 h, and decline with a half-life of 19 h once inflammation resolves. While CRP is a reliable marker of inflammation, it lacks specificity for sepsis, as elevated CRP levels occur in multiple inflammatory diseases [32,33]. The slow rise in CRP levels also limits its utility for early sepsis identification. However, CRP is helpful for monitoring disease progression and response to treatment due to its rapid decline when inflammation subsides. A meta-analysis of studies through 2008 found that CRP’s sensitivity for differentiating bacterial from non-bacterial infection was 77%, with a specificity of 79% [39]. Although CRP’s accuracy for early sepsis diagnosis is debated, it has shown promise as a prognostic indicator, with studies reporting higher mortality in children whose CRP levels failed to decline after initial treatment [40,41,42,43].

#### 3.1.4. Procalcitonin (PCT)

Procalcitonin (PCT), a precursor of calcitonin, is typically undetectable in healthy individuals but increases rapidly in bacterial infections due to widespread CALC-1 gene expression, triggered by proinflammatory cytokines [44,45]. Viral infections, which often stimulate interferon-γ, generally do not induce significant PCT elevation, making PCT a potential tool for distinguishing bacterial from viral infections [46,47,48]. Despite these strengths, clinical studies in pediatric sepsis have shown mixed results. While PCT levels correlate with bacterial sepsis severity and have better diagnostic accuracy than CRP, they are insufficient as standalone markers because of the variability across different inflammatory conditions and study populations [7,49,50,51,52,53,54,55,56]. For sepsis detection, PCT’s sensitivity at a threshold of 0.5 ng/mL was reported at 55%, with a specificity of 85%; at 2 ng/mL, the sensitivity was 30% with a 95% specificity [49]. However, PCT is valuable in disease monitoring, with serial PCT measurements indicating prognosis; for instance, rapid PCT decline after treatment initiation is associated with lower mortality [57,58,59].

#### 3.1.5. Ferritin

Ferritin (FE), an iron-storage protein, is elevated in response to inflammation due to cytokine stimulation, including IL-6 and TNF-α [60]. While high FE levels have been observed in pediatric sepsis, its increase is nonspecific, seen in various infectious and non-infectious conditions [61,62,63]. Elevated FE may indicate severe inflammation rather than early infection, limiting its role in sepsis diagnosis or real-time treatment adjustments. However, FE levels correlate with sepsis severity, with studies indicating higher risks of ICU admission and mortality in children with FE > 3000 ng/mL [64,65].

#### 3.1.6. Lactate

Lactate production rises in response to tissue hypoxia and hypoperfusion, conditions common in sepsis-induced shock. Persistent high lactate levels correlate with sepsis severity and increased mortality, making it a useful prognostic marker [66,67]. Rapid lactate reduction (>10% within 4 h of admission) is associated with improved outcomes, reflecting improved perfusion [68]. However, lactate’s use in early sepsis detection is limited since it indicates hypoperfusion rather than infection onset. Moreover, venous lactate can be falsely elevated, necessitating arterial confirmation to ensure accuracy in prognostic assessment [69].

### 3.2. New Biomarkers

In recent years, several new biomarkers theoretically capable of improving early definition of immediate and long-term prognosis of sepsis have been identified. Some of them are potentially effective as they take into consideration early significant sepsis-induced organ dysfunctions. As septic patients with significant functional impairment of one or more organs have an increased risk of morbidity and motility, the evidence that blood levels of some physiological variables are modified when organ dysfunction develops and gets worse may allow to consider these indicators as reliable biomarkers of sepsis development, severity and prognosis [70]. Other new biomarkers have been chosen taking into account the modification of immune system activity that occur during sepsis development and course and the gene expression-based endotypes of septic shock. In both cases, the use of these biomarkers allowed for obtaining reliable information on sepsis outcome [71].

#### 3.2.1. Biomarkers Useful for an Early Identification of Organ Dysfunctions

##### Kidney Injury

Sepsis-associated acute kidney injury (AKI) can be diagnosed in up to half of all critically ill children with septic shock and lead to death in up to 70% of the cases [72]. As blood urea nitrogen and serum creatinine concentrations, generally used to evaluate kidney function in clinical practice, do not promptly reflect AKI [73], other markers have been studied to evaluate early kidney dysfunction in children with sepsis.

Neutrophil Gelatinase-Associated Lipocalin (NGAL) is an ion-transporting agent produced in the distal nephron whose synthesis is up-regulated in response to kidney injury [45]. It can be found in both serum and urine 48 h before the increase in blood urea nitrogen and serum creatinine and the reduction in urine output. For this, NGAL can be considered, at least theoretically, a better marker of early AKI than traditional marker of AKI. An early modification of NGAL concentration in children with sepsis had already been reported by Wheleer et al. some years ago [74]. These authors measured NGAL levels during the first 24 h of admission to the PICU and found they were significantly higher in children with septic shock (median 302 ng/mL, IQR 151–570 ng/mL) than in healthy children (median 80 ng/mL, IQR 55.5–85.5 ng/mL; *p* < 0.001). More detailed data on the role of NGAL in children with sepsis were more recently reported in a study enrolling 65 children with sepsis and 20 healthy controls matched for age and sex [75]. NGAL concentrations were evaluated within 24 h and after 72 h of hospital admission and found increased in proportion to the severity of renal impairment measured through the Pediatric Risk, Injury, Failure, Loss, End Stage Renal Disease (pRIFLE) criteria [76]. A value of 40 ng/mL within the first 24 h of hospitalization was found to be highly specific and sensitive for predicting AKI and for grading its severity, with a sensitivity of 90.9% and specificity of 75.8%. However, data collected after 3 days of admission revealed that serum creatinine values of 0.7 mg/dL had greater sensitivity (95.5% vs. 86.4%) and specificity (95% vs. 90%) than NAG values in identifying AKI. This highlights that whether NGAL could be significantly effective in the early identification of AKI in children with sepsis, serum creatinine level can allow a more precise evaluation of AKI evolution.

##### Neurological Damage

As severe encephalopathy is common in patients with sepsis, early detection of neurological involvement may allow identification of at-risk cases and prescription of effective preventive and therapeutic measures. Although medical examination, possibly associated with appropriate imaging, is essential for identification of neurological damage in subjects with undefined clinical conditions suggesting sepsis, useful information in this regard may derive from the evaluation of circulating levels of adipocyte fatty-acid-binding protein (A-FaBP). This is an intracellular lipid-binding protein that plays a relevant role in mediating intracellular fatty acid trafficking [77], in glucose metabolism and inflammation development [78]. A-FaBP has been found increased in adult patients with conditions associated to neurological damage, such as severe atherosclerosis, cardiovascular disease events, ischemic stroke, and sepsis suggesting its potential use for an early identification of sepsis in pediatric patients [79]. This supposition was confirmed in a study enrolling 30 children with sepsis or septic shock and two controls groups, the first with 30 healthy subjects and the second with 30 febrile children without clinical manifestations suggesting sepsis [80]. The results have shown that A-FaBP levels measured in pooled serum samples from days 1 and 2 after ICU admission could discriminate patients with sepsis and early neurologic damage. They were significantly increased in patients with neurological dysfunction compared to patients without (29.3 ng/mL, interquartile range [IQR] 17.2–54.6 vs. 14.6 ng/mL; IQR 13.3–20.6; *p* < 0.05). Further information regarding mortality risk in children with sepsis may derive from the evaluation of insulin-like growth factor 1 (IGF-1) and insulin-like growth factor binding protein 3 levels (IGFBP-3). Comparing levels of these factors in children with sepsis according to the outcome of disease, it was found that both were significantly lower in non survivors than in survivors. The greatest difference was found for IGFBP-3. A reduction at any time after day 3 of illness resulted in 35-fold increased odds of death (OR 35; 95% CI 1.6–750; *p* = 0.004)

##### Respiratory Damage

In children with sepsis development of acute respiratory distress syndrome (ARDS) can occur with risk of mortality in 11% of cases [81]. Characterizing the degree of lung injury in pediatric acute respiratory distress syndrome. True incidence of this condition in these patients can vary from 9% [82] to 83% [83], according to the definition used for ARDS. However, it is a very severe condition that must be identified early to avoid rapid negative evolution. ARDS can be due to direct or indirect damage to the lung depending on whether the infection is pulmonary or extrapulmonary origin. In direct cases, ARDS depends on epithelial damage, whereas in indirect ARDS respiratory damage arises from endothelium activation [84].

In children with direct ARDS, several potential biomarkers have been identified. Krebs von den Lungen-6 (KL-6), Clara cell protein 16 (CC16), soluble receptor for advanced glycation end products (sRAGE), and soluble intercellular adhesion molecule (sICAM) are specific epithelium proteins that have been found to be increased in several pediatric respiratory diseases associated with severe epithelial damage [84]. They have shown promise as biomarkers of early ARDS in children with sepsis and may have value as predictive and prognostic factors. However, studies in children with sepsis and severe respiratory involvement are very few and definitive conclusions cannot be drawn [85,86,87]. Among them, the best studied in pediatrics is KL-6 that is a high-molecular-weight glycoprotein that is present on alveolar type II pneumocytes. Higher serum levels of this protein have been found in children with several severe respiratory diseases, including those with sepsis and ARDS. Moreover, bad prognosis was more frequent in those with highest KL-6 values [88].

Regarding indirect ARDS, it has been shown that, in children with sepsis, endothelial activation is accompanied by a relevant increase in some biological factors, including angiopoietin-2, angiopoietin-2/angiopoietin-1 ratio, vascular cell-adhesion molecule, and von Willebrand factor. Moreover, persistent biomarkers changes would be associated with poor disease outcome. These findings suggest that these factors can be considered early biomarkers of ARDS development and prognostic factors for negative outcome of pediatric sepsis [89]. Data collected in 33 children have later confirmed this hypothesis [90]. It was evidenced that a model using the previously cited biomarkers was more effective than a model using only clinical variables in identifying indirect ARDS. The biomarkers had 89% (95% CI 80–97) sensitivity, 80% (95% CI 69–92) specificity, positive predictive value (PPV) 84% (95% CI 74–93), and negative predictive value (NPV) 86% (95% CI 76–96) [90].

Finally, risk of death from ARDS can be better defined using PARDSEVERE [91]. This is an adaptation of PERSEVERE and PERSEVERE-II predicting models that had been found to have poor predictive ability for mortality of children with sepsis and ARDS. PARDSEVERE included only chemokine ligand 3 (CCL3), heat shock protein 70 kDa 1B (HSPA1B), interleukin-8 (IL-8), and age. Compared to PERSEVERE and PERSEVERE-II that had AUROC curves of 0.61 (95% CI 0.49–0.73) and 0.76 (95% CI 0.65–0.86; *p* = 0.029), respectively, PARDSEVERE had an AUROC of 0.85 (95% CI 0.78–0.92; *p* < 0.001 and *p* = 0.053 compared with PERSEVERE and PERSEVERE-II) [91].

#### 3.2.2. Biomarkers Based on Evaluation of Immunity and Gene Expression-Based Endotypes

Using genome-wide expression profiling, a panel of candidate stratification gene probes to predict outcome of children with septic shock was identified. Initially, twelve genes coding proteins with known biological mechanisms possibly associated with outcomes from septic shock were identified. Later, only five biomarkers [IL-8, CCL3, HSPA1B, granzyme B (GZMB), and matrix metalloproteinase 8 (MMP-8)] together with the age of the patient were considered, resulting in a model named PERSEVERE that was repeatedly tested and considered effective for the identification of a certain number of children with septic shock at high risk of mortality within the first 24 h of sepsis diagnosis [92]. More recently, PERSEVERE was recalibrated with the inclusion of the admission platelet count as an additional predictor variable [93].

A study evaluated 220 unselected children with septic shock during the first 24 h of admission to the intensive care unit [94]. PERSEVERE-II was used to predict 28-day all-cause mortality. The sensitivity was 93% (95% CI 79–98), the specificity was 74% (95% CI 69–79), the PPV was 32% (95% CI 24–41), and the NPV was 99% (95% CI 96–100).

Moreover, in some children PERSEVERE II was found effective in the early identification of specific organ damage, frequently associated with poor prognosis. In children with septic shock, the PERSEVERE-II biomarkers predicted severe, sepsis-associated AKI on day 3 (D3 SA-AKI) from disease development [95]. The increase in PERSEVERE-II mortality probability was independently associated with increased odds of severe D3 SA-AKI (OR, 1.4; 95% CI 1.2–1.7; *p* < 0.001). Moreover, a strict correlation was found between low PERSEVERY-II biomarkers and increased odds of renal recovery among patients with early AKI, highlighting the potential use of this model to identify cases with good prognosis.

PERSEVERE II was also found effective in early recognition of sepsis-associated myocardial dysfunction (SAMD). Myocardial dysfunction is a potentially fatal complication of sepsis. It has been shown that more than half of the children (54.5%) with sepsis-induced myocardial dysfunction (SMD) and shock die compared to 7.5% of those without (*p* < 0.001). Consequently, SMD is considered an independent predictor of mortality [96]. Accurate cardiac function evaluation [97,98], eventually associated with lactate [99] or ferritin levels [100], can carefully predict mortality. However, early recognition of SAMD seems particularly important as early targeted clinical management may shorten time to reversal of shock. In a group of 181 children with septic shock, among whom 32 had SAMD, it was shown that PERSEVERE II biomarkers estimated SAMD risk with an AUROC of 0.90 (95% CI 0.85–0.95).

However, both PERSEVERE and PERSEVERE-II seem effective only for prediction of mortality in children admitted to the PICU with septic shock. They cannot estimate the risk of deterioration among non-PICU patients suspected of being bacterially infected at the time of suspicion for infection. This was evidenced in a cohort of largely general-ward-based, immunocompromised pediatric patients. The ability of PERSEVERE and PERSEVERE-II to predict mortality at 28 days was a poor predictor of mortality at 28 days in this cohort of patients who are immunocompromised. As with clinical deterioration, the AUROCs were low at 0.62 (95% CI 0.46–0.79, *p*-value 0.06) and 0.67 (955 CI 0.55–0.79, *p*-value 0.008) [101]. However, it was evidenced that considering together three (IL8, HSPA1B, and CCL3) of the five biomarkers included in PERSEVERE-II, with the additions of age and platelet count, it was possible to develop a new model with high ability to identify clinical deterioration or death from bacterial infection in children. The AUROC of the new model to predict 28-day mortality was 0.87 (0.80–0.94, *p*-value < 0.0001).

These findings highlight that models like PERSEVERE and PERSEVERE-II, if developed for the identification of the outcome of sepsis in a well-defined group of children, should be validated when used in patients with different characteristics. Moreover, data collected with these models suggest that investigating immune system function in children with sepsis can offer early information on diagnosis and disease evolution and outcome. Sepsis affects various immune cell functions. For example, in patients with sepsis a relevant imbalance between proinflammatory and anti-inflammatory cytokines occurs, and this is considered a relevant factor in the determination of multiorgan dysfunction. Monitoring IL-10 plasma levels might be useful for identifying children with severe multiorgan dysfunction and poor prognosis. This biomarker has been found to be higher in children with three or more organ failures compared to children with less than three organ failures (days 1 and 3; *p* < 0.05). Moreover, nonsurvivors had higher IL-10 levels (day 3; *p* < 0.05) [102]. Increases in other cytokines such as IL-8 [103] and IL-27 [104] have been reported in several studies that enrolled children with sepsis, suggesting that their monitoring alone or in combination with other biomarkers may be of value in the evaluation of children with sepsis. However, studies of immune systems in sepsis are still in a relatively early stage, and conclusions on their role in pediatric sepsis evaluation cannot be drawn.

## 4. Conclusions

The identification and management of pediatric sepsis has long been an important area of research. However, despite substantial efforts, early detection and risk stratification remain challenging. In the initial stages, sepsis often presents with symptoms indistinguishable from mild, self-limiting infections or chronic conditions, complicating timely diagnosis. While various early warning scores have been developed, their utility is limited, particularly in prehospital settings, where their predictive accuracy often falls short. Traditional biomarkers, such as PCT, have contributed to refining diagnostic accuracy, but they are insufficient as standalone indicators; many cases go undetected, leading to both antibiotic overprescription and the risk of delayed treatment in children who may progress to severe sepsis.

The combined use of multiple biomarkers has shown promise in improving early screening, although this approach may be less feasible outside hospital settings due to complexity and resource requirements. A less-explored area is the prediction of sepsis outcomes to tailor therapy according to the individual trajectory of each child’s illness. While serial measurements of traditional biomarkers throughout the disease course can provide some prognostic information, their reliability remains limited, and therapeutic decisions often depend on clinical judgment rather than biomarker guidance.

Novel biomarkers that enable the early identification of sepsis-related organ dysfunction show potential for better prognostic and predictive insights in pediatric sepsis. However, despite recent advancements, significant hurdles remain. Most novel biomarkers are currently available only in hospital settings, some require further validation, and others involve specialized assays not routinely accessible in standard laboratories, limiting their widespread clinical adoption. Further research is essential to address these challenges, establish reliable protocols, and facilitate broader use in diverse healthcare settings.

Until these solutions are available, it is crucial that healthcare providers continue to integrate clinical tools and biomarkers thoughtfully with clinical judgment. A multifaceted approach combining these elements will likely offer the best support for identifying and managing pediatric sepsis, optimizing outcomes while minimizing risks.

## Figures and Tables

**Table 1:** Comparison of Pediatric Early Warning Scores (PEWS) and their applications in sepsis diagnosis.

PEWS Tool	Key Indicators Assessed	Strengths	Limitations	Settings
National PEWS	Vital signs (e.g., heart rate, respiratory rate), behavior, oxygen saturation	High specificity for ICU admissions, good prediction for critical care needs	Limited sensitivity, especially in non-severe cases	Primarily hospital EDs
Brighton PEWS	Vital signs, signs of respiratory distress, mental status	Easy to use, high specificity for certain outcomes	Low sensitivity for severe illness identification	Hospital-based
COAST PEWS	Vital signs, oxygen requirements, mental status	Simple scoring, effective in some hospital-based studies	Poor accuracy in prehospital settings	Mostly hospital, limited in prehospital
Bedside PEWS	Similar to National PEWS with bedside integration	High discriminative value, user-friendly	Limited scope in predicting long-term outcomes	Emergency departments, bedside
Modified PEWS with EHR integration	Continuous monitoring with automated alerts	Improved speed and accuracy, reduced errors	Requires hospital EHR setup	Hospital settings only

**Table 2:** Summary of traditional and novel biomarkers in pediatric sepsis diagnosis and prognosis.

Biomarker	Type	Key Applications	Advantages	Limitations
**Procalcitonin (PCT)**	Traditional	Diagnosis, bacterial infection indicator	Differentiates bacterial from viral infections, moderate prognostic value	Lacks sensitivity for early sepsis diagnosis
**C-Reactive Protein (CRP)**	Traditional	Inflammation marker	High availability, useful for monitoring treatment response	Nonspecific, elevated in many conditions
**Neutrophil Gelatinase-Associated Lipocalin (NGAL)**	Novel	Early kidney injury detection	Early acute kidney injury detection, higher sensitivity than creatinine	Limited availability, less studied in children
**Adipocyte Fatty-Acid-Binding Protein (A-FaBP)**	Novel	Neurological dysfunction prediction	May indicate early neurological damage in sepsis	Requires further validation in pediatric cases
**Krebs von den Lungen 6 (KL-6)**	Novel	Lung injury	Specific for respiratory epithelial damage, prognostic for respiratory failure	Limited pediatric studies, mainly in hospitals
**Interleukin (IL)-10, IL-8, and IL-27**	Novel, Immune-based	Immune function assessment, severity prediction	Correlate with sepsis severity, potential prognostic markers	Complex interpretation, variability among patients
Ferritin	Traditional	Inflammation assessment	Potential use for real-time treatment adjustments	Poor role in early diagnosis
Lactate	Traditional	Marker of tissue hypoxia and hypoperfusion	Values correlate with sepsis severity and mortality risk	Limited value for early diagnosis

## Data Availability

Not applicable.
